# Dopamine-grafted heparin as an additive to the commercialized carboxymethyl cellulose/styrene-butadiene rubber binder for practical use of SiO_x_/graphite composite anode

**DOI:** 10.1038/s41598-018-29705-y

**Published:** 2018-07-27

**Authors:** Kukjoo Lee, Sanghyun Lim, Nakgyu Go, Jaemin Kim, Junyoung Mun, Tae-Hyun Kim

**Affiliations:** 1 Organic Material Synthesis Laboratory, Department of Chemistry, Incheon, Korea; 2 Research Institute of Basic Sciences, Incheon National University, 119 Academy-ro, Songdo-dong, Yeonsu-gu Incheon, 406-772 Korea; 3 Department of Energy and Chemical Engineering, Incheon National University, 119 Academy-ro, Songdo-dong, Yeonsu-gu Incheon, 406-772 Korea

## Abstract

Graphite is used commercially as the active material in lithium ion batteries, frequently as part of a graphite/SiO_x_ composite. Graphite is used in conjunction with SiO_x_ to overcome the limited energy density of graphite, and to lessen the adverse effects of volume expansion of Si. However, electrodes based on graphite/SiO_x_ composites can be made with only 3–5 wt % SiO_x_ because of the increased failure of electrodes with higher SiO_x_ contents. Here, we developed a new polymer binder, by combining dopamine-grafted heparin with the commercial binder carboxymethyl cellulose (CMC)/styrene butadiene rubber (SBR), in order to more effectively hold the SiO_x_ particles together and prevent disintegration of the electrode during charging and discharging. The crosslinking using acid-base interactions between heparin and CMC and the ion-conducting sulfonate group in heparin, together with the strong adhesion properties of dopamine, yielded better physical properties for the dopamine-heparin-containing CMC/SBR-based electrodes than for the commercial CMC/SBR-based electrodes, and hence yielded excellent cell performance with a retention of 73.5% of the original capacity, a Coulombic efficiency of 99.7% at 150 cycles, and a high capacity of 200 mAh g^−1^ even at 20 C. Furthermore, a full cell test using the proposed electrode material showed stable cell performance with 89% retention at the 150^th^ cycle.

## Introduction

Lithium-ion secondary batteries are being intensively studied due to their potential uses as large-scale energy storage devices in applications such as electric vehicles as well as energy storage systems^1–4^. However, currently available lithium-ion secondary batteries use graphite as the anode material, and as a result do not have sufficient energy density to efficiently power such high-energy applications.

Much effort is hence being devoted to increasing the energy density levels of lithium-ion batteries. To achieve this, materials based on Li alloys, such as lithium-tin and lithium-silicon, have attracted much recent attention as alternate anode materials due to their high capacities^5–9^. For example, the silicon electrode has a high theoretical capacity, more than ten times greater than that of the currently used anode material for lithium-ion batteries (i.e., graphite), which has a theoretical capacity of only about 372 mAh g^−1^. However, silicon has inherent problems due to volume expansion and shrinkage of about 400% during the charge-discharge process of Si + 4.4Li ⇆ Li_4.4_Si^5,6^. When such changes in volume are repeated, mechanical stresses build up that damage the anode, induce its components (e.g., conducting agents and binders) to separate from one another, and cause loss of electrical pathways by isolating Si particles having insulation properties, leading to rapid deterioration of the battery system^9–12^. Thus, many researchers have examined modified forms of silicon, for example, nano-sized particles, in order to reduce the stress caused by the volume expansion of Si^13,14^. The preparation of these nano-architectured silicon anode materials, however, inevitably increases the production costs.

Silicon oxides (SiO_x_), on the other hand, are relatively inexpensive to produce and have been commonly used as composite materials with Si to lessen the adverse effects of the volume expansion of Si. In these composites, either crystalline or amorphous Si cores are uniformly dispersed in a SiO_2_ matrix, and the stable lithium-silicates formed after lithiation of this material have been shown to reduce the magnitude of the changes in the volume of Si during cycling^12,15–17^. Indeed, in currently used and commercialized active materials based on graphite, replacing some of the graphite with SiO_x_ has been found to be the most practical way of overcoming the limited energy density of the graphite. The SiO_x_ content of the active material, however, has been limited to at most 3–5 wt.% due to the rapid increase in electrode failure with increasing SiO_x_ content as a result of the above-mentioned side reactions of Si.

Aside from the problems directly related to the silicon itself, developing a strong polymer binder that can endure large volume changes of Si and prevent electrodes from rupturing has also been widely pursued recently. Polymers such as poly(acrylic acid) (PAA) that consist of many carboxylic acid functional groups^18,19^ and polysaccharides such as alginates^20,21^, Xanthan gum^22^ and Pullulan^23^ have been shown—due to their excellent physical properties resulting from their strong interactions with OH groups on silicon particles—to be of use for increasing the lifespan of Si anodes. These polymers may thus be good candidates as polymeric binders for Si as well as other high-capacity anode materials such as Sn and Ge, and may even be a viable substitute for carboxymethyl cellulose (CMC)/styrene butadiene rubber (SBR), the binder currently used in commercial graphite anodes. In addition, cross-linkages via covalent bonds^24–26^ or even noncovalent ones such as hydrogen bonds^27–31^ have been exploited to further improve the physical properties of these polymer binders, yielding markedly improved cell performance.

We report herein a new polymer binder based on dopamine-functionalized heparin/CMC/SBR, in which dopamine-grafted heparin was used as an additive in the commercially available CMC/SBR binder, and show that it improves the performance of SiO_x_/graphite composite-based anodes. Heparin is a highly sulfonated, biocompatible, water-soluble, and naturally derived polysaccharide. It has various functional groups, which have contributed to its effectiveness and hence wide use as an anticoagulant and inhibitor of both angiogenesis and tumor growth^32^. Although heparin is difficult to apply directly as a binder to Si anodes due to its poor physical properties when used alone^33^, the many functional groups of heparin allow it to interact with CMC. These functional groups include amines, which can reversibly interact with the acid groups in CMC through hydrogen bonding, and sulfonates, which can further contribute to Li^+^ conduction. In addition, dopamine, inspired by its role as an adhesive in mussel foot protein, was further incorporated onto heparin due to dopamine’s well-known excellent adhesion to various surfaces including Si anodes^34^. Therefore, we produced dopamine-functionalized heparin and added it to the CMC/SBR binder in order to (1) mechanically strengthen this binder by crosslinking, (2) improve the adhesion property of the corresponding binder to Si and hence suppress the volume expansion of Si, and (3) endow the crosslinked binder system with the ability to conduct ions.

## Results

### Electrochemical performance and failure mode analyses

**Figure 1:**
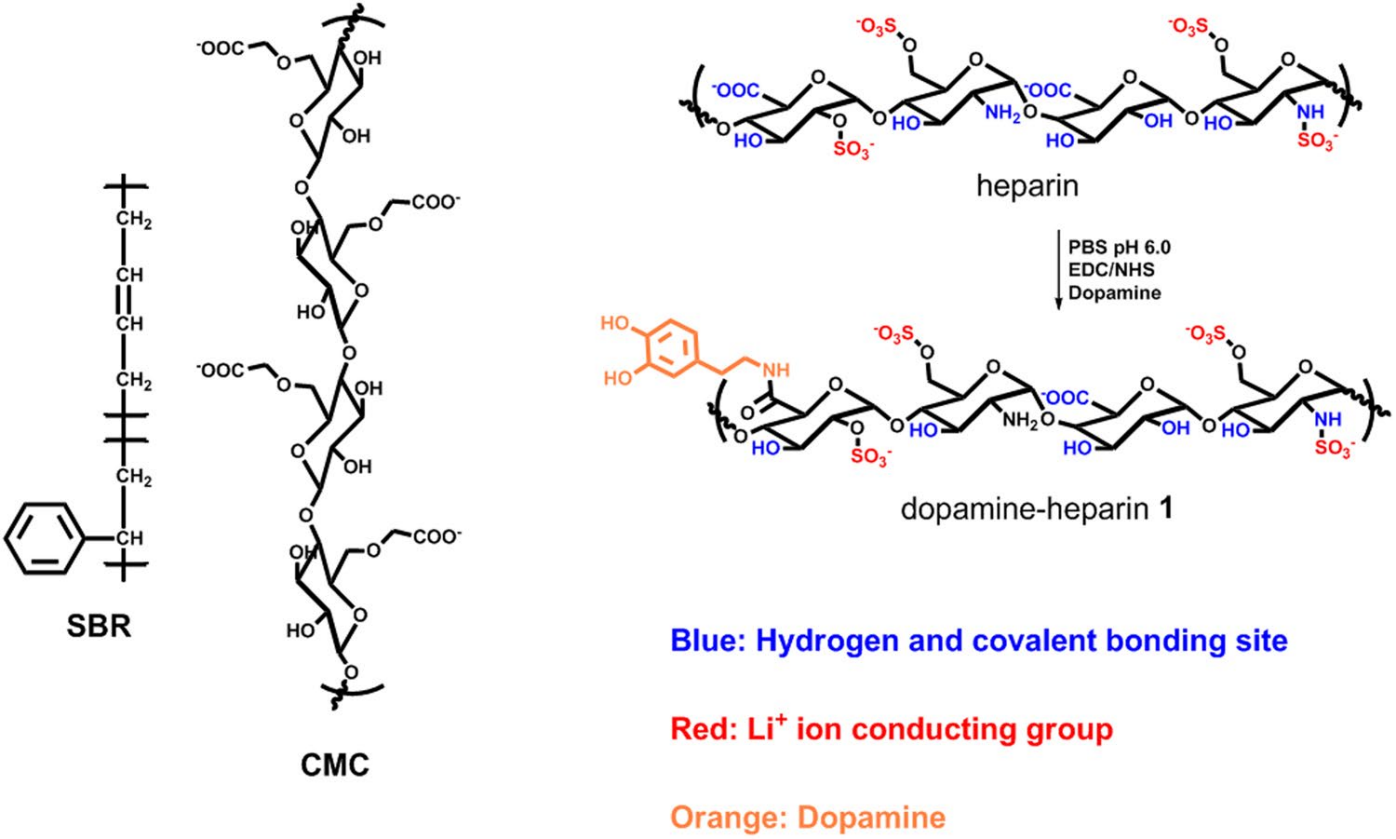
Preparation of the dopamine-heparin binder **1**.

The addition of dopamine to heparin was accomplished via the N-(3-dimethylaminopropyl)-N′-ethylcarbodiimide hydrochloride (EDC) coupling reaction, as shown in Fig. 1. To prevent the oxidation of dopamine, the pH was maintained at 6.0 using a phosphate buffer solution (PBS) while the reaction proceeded.

**Figure 2:**
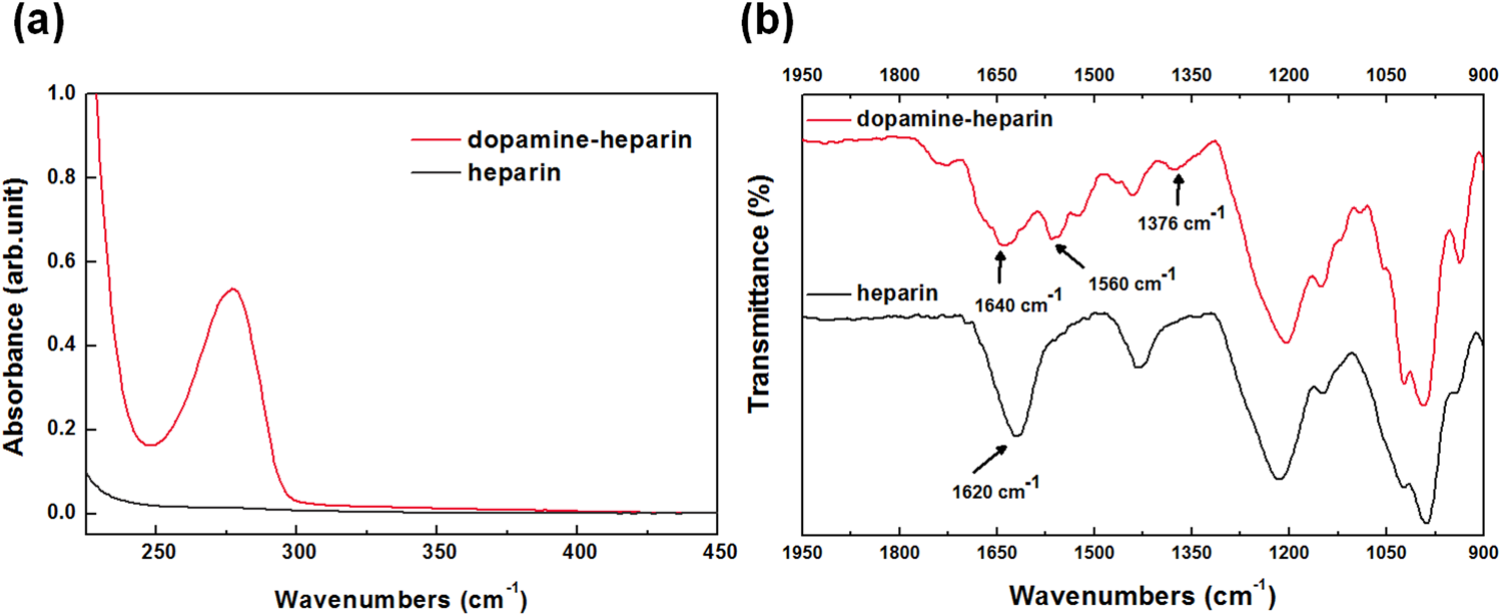
UV-Vis spectra (**a**) and FT-IR spectra (**b**) of dopamine-grafted heparin **1** and heparin.

A structural analysis of the dopamine-functionalized heparin (dopamine-heparin, **1**) was carried out by performing UV-Vis and FT-IR spectroscopy investigations. The UV-Vis spectrum of **1** (Fig. 2a) showed a characteristic absorption peak at a wavelength of about 280 nm, confirming that the catechol moiety of dopamine was successfully grafted onto heparin^32,34^. In addition, the absence of any additional peaks at wavelengths greater than 300 nm indicated that no undesired oxidation of dopamine occurred^32^. The amount of dopamine incorporated was measured to be 11 wt.% of the total weight of dopamine-heparin **1**, as determined by carrying out a UV-Vis spectroscopic quantitative analysis using dopamine standard solutions (see Supplementary Fig. S1)^32^.

Further analyses of **1** using FT-IR spectroscopic investigations confirmed the structure (Fig. 2b): the C = O stretching peak of heparin at 1620 cm^−1^ shifted to 1640 cm^−1^ after conjugation of dopamine due to the formation of amide bonding. In addition, a peak corresponding to the secondary amide NH bending appeared at 1560 cm^−1 ^^32,35^, indicating that an amide bond successfully formed between the dopamine and heparin. Furthermore, a new peak corresponding to the aromatic OH stretching of the catechol moiety appeared at 1376 cm^−1^.

### Preparation of electrodes and analyses of the mechanical properties

Electrodes were fabricated using a composite of SiO_x_ and graphite as the active material, Super-P as the conducting agent, and CMC/SBR, heparin/CMC/SBR or dopamine-heparin/CMC/SBR as the polymer binder in a ratio of active material: conducting agent: binder of 80:10:10 by weight. The weight ratio of CMC to SBR was 1:2, and 10 wt.% of heparin or **1** relative to CMC was added to CMC/SBR for the fabrication of the heparin/CMC/SBR- or dopamine-heparin/CMC/SBR-based electrode, respectively. The results were compared with those obtained using the electrode containing only CMC/SBR as the polymer binder.

**Figure 3:**
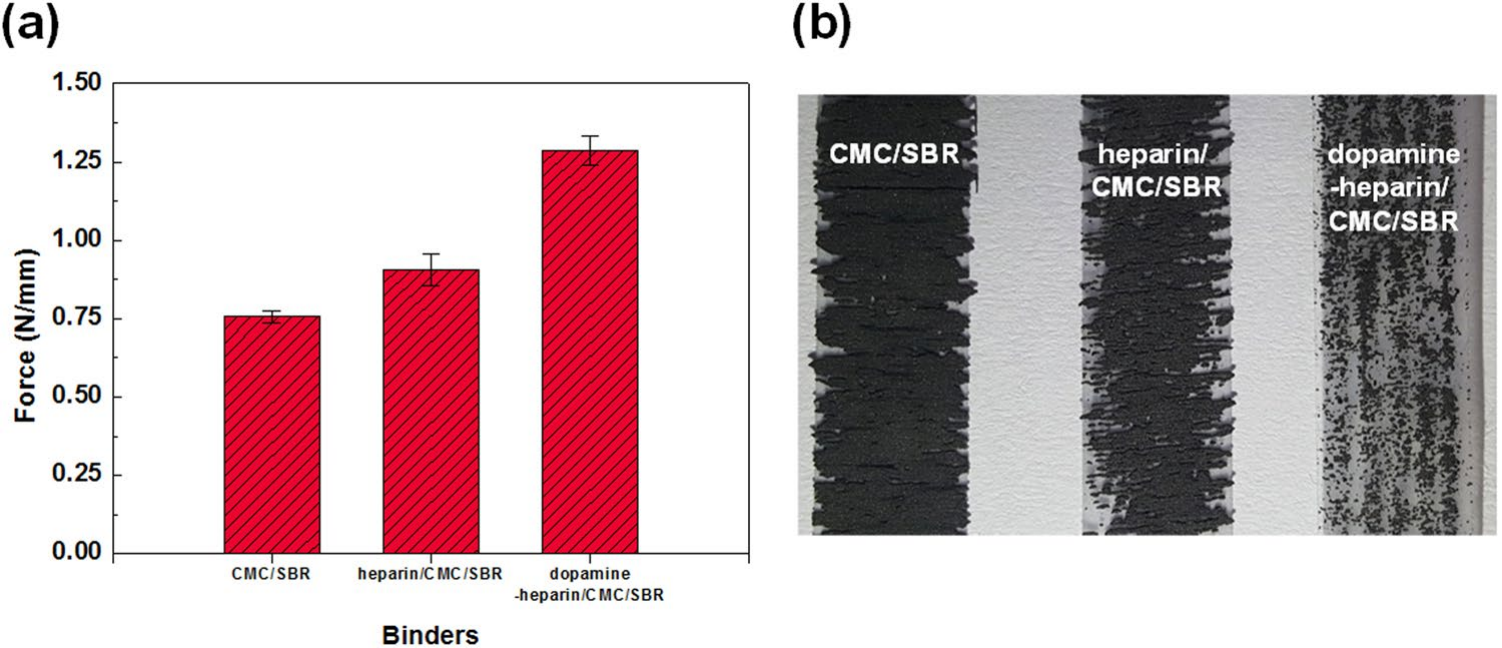
Peel test results with its mean forces (**a**) and images (black regions represent the detached electrode components and the white regions indicate transmitted light) (**b**) of the CMC/SBR-, heparin/CMC/SBR- and dopamine-heparin/CMC/SBR-based electrodes.

The binding affinities for the three electrodes (CMC/SBR, heparin/CMC/SBR and dopamine-heparin/CMC/SBR) were then investigated by carrying out 180-degree peel tests (Fig. 3a). In the case of the SiO_x_/graphite electrode composed of the pristine CMC/SBR polymer binder, an average binding affinity of 0.757 N was obtained, while this value increased to 0.907 N when heparin was added to CMC/SBR (heparin/CMC/SBR electrode). A dramatic increase in the binding affinity was further obtained for the electrode with the CMC/SBR binder containing dopamine-heparin (dopamine-heparin/CMC/SBR), with a measured value of 1.287 N. These results suggested that the addition of heparin to CMC/SBR mechanically strengthened the electrode due to the formation of physical interactions between CMC and heparin, and that the durability of the electrode in the presence of mechanical stress was further increased by the strong adhesion resulting from the inclusion of dopamine in the dopamine-grafted heparin (dopamine-heparin, **1**)^27,34^.

We further used an optical microscope to examine the surface of the tape detached from the electrode after the peel test to determine whether the addition of dopamine-heparin (to form a dopamine-heparin/CMC/SBR binder) reduced the amount of Si exfoliated from the electrode. As shown in Fig. 3b, the amount of the electrode material detached from the current collector was inversely related to the adhesive force generated by the binder in Fig. 3a^28^. The tapes pulled off the pristine CMC/SBR-based electrode were stained with a lot of slurry and transmitted little light, indicating that much of the Si material in these cases became detached from the electrode and transferred to the tape. In contrast, the tapes pulled off the heparin/CMC/SBR-based electrode were stained with only a very small amount of slurry, and were observed to transmit a considerable amount of light, indicative of the better ability of heparin/CMC/SBR than of pristine CMC/SBR to prevent material from being detached from the electrode and consistent with the stronger adhesion force of the heparin/CMC/SBR binder based on the peel test. And this trend continued with the inclusion of dopamine: tapes pulled off the dopamine-heparin/CMC/SBR-based electrode, showed the least amount of slurry and transmitted the most light, consistent with the dopamine-heparin/CMC/SBR binder having displayed an adhesion force greater than those of the other binders.

### Electrochemical properties

**Figure 4:**
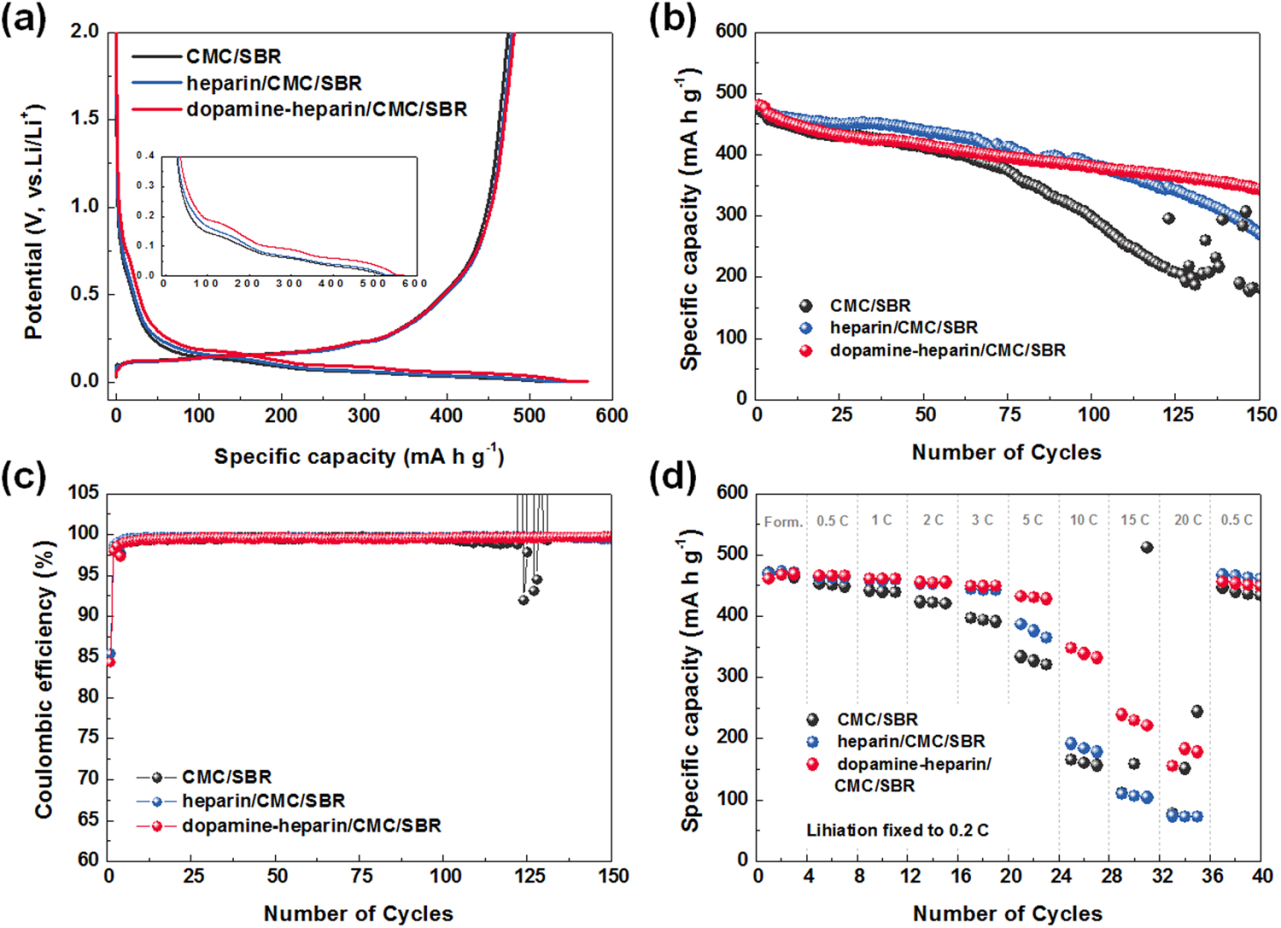
The 1^st^ cycle voltage profiles (**a**), cyclability (**b**), the Coulombic efficiencies (**c**) and rate capability (**d**) of the SiO_x_/graphite composite anodes with various binders.

After investigating the mechanical properties of the electrodes containing the heparin/CMC/SBR and dopamine-heparin/CMC/SBR binders, we then assessed the electrochemical performances of their corresponding cells. We performed the electrochemical evaluations at a low rate (specifically with a discharge rate of 0.2 C and charge rate of 0.5 C, where 1 C equals 450 mA g^−1^), and compared the results with the cell performance of the pristine CMC/SBR-based electrodes (see Fig. 4).

The voltage profiles of the prepared cells during the 1^st^ cycle showed similar trends irrespective of the binder (Fig. 4a), suggesting that the addition of heparin or dopamine-heparin (**1**) did not cause any particular side reactions. In addition, the voltage profiles of both the heparin- and dopamine-heparin-based electrodes showed lower resistance during lithiation than did the electrode prepared from CMC/SBR. The enhanced physical properties of the heparin and dopamine-heparin binder systems have been suggested to result in less polarization due to the reduction of the contact resistance of the electrode^36^. Having the highest mechanical strength, the dopamine-heparin-based electrode indeed showed the lowest resistance.

In addition, the specific discharge capacities of the cells prepared using the three binders were also determined in order to assess the effect of the binder on cyclability (Fig. 4b). The specific capacity of the CMC/SBR-based electrode started to deteriorate rapidly after 50 cycles, and at 100 cycles showed a value of 286 mAh g^−1^ and a retention of 62.3% of the original specific capacity. After about 100 cycles, this electrode showed particularly poor cycling stability. This poor stability was attributed to the CMC/SBR binder, with its poor adhesion properties, no longer being able to accommodate the repeated changes in the volume of the silicon electrode. The cell performances of the electrode made of the heparin/CMC/SBR binder and that made with the dopamine-heparin/CMC/SBR binder, however, were much better than the cell performance of the electrode made with the pristine CMC/SBR binder. The superior performance of the former two cells was attributed to their enhanced physical properties. A specific capacity of 380 mAh g^−1^, representing a retention of 81.3% of the original capacity, was observed at 100 cycles, and a specific capacity of 268 mAh g^−1^ and retention of 57.3%, were observed at 150 cycles for the electrode including heparin/CMC/SBR. The dopamine-heparin/CMC/SBR-based binder system showed the best cell performance. Although the specific capacity and retention values of the dopamine-heparin/CMC/SBR-based binder system at 100 cycles, with values of 378 mAh g^−1^ and 81.3%, respectively, were similar to those of the heparin/CMC/SBR-based system, a much greater reversible capacity of 343 mAh g^−1^ and retention of 73.5% were obtained at 150 cycles for the dopamine-heparin/CMC/SBR-based system than for the heparin/CMC/SBR-based system. Since the three cells were subjected to the same conditions, the superior specific capacity and percent retention of original specific capacity up to the 150 cycles for the cell having the dopamine-heparin/CMC/SBR binder were therefore attributed mainly to the superior adhesion properties of this binder material.

The dopamine-heparin-CMC/SBR electrode also displayed excellent Coulombic efficiency (Fig. 4c). It displayed an initial Coulombic efficiency (after the formation cycles) of 97.4%, a value of 98.9–99.5% at the 20^th^ cycle, and very high value of 99.7% at the 150^th^ cycle. These values were higher than those of the CMC/SBR- and heparin/CMC/SBR-based electrodes.

We also performed rate capability tests for the three types of electrode (Fig. 4d). Their 0.1 C-capacities were similar to those in the formation stage of the cycling performance experiment. The CMC/SBR-based electrode (having the worst mechanical properties) showed a much larger decay of specific capacity as the C rate was increased; the performance of this electrode indicated that it would be difficult to use above 15 C. The heparin-based electrode, in contrast, exhibited a stable capacity up to 3 C. Although a constant capacity loss occurred when the rate was increased to above 3 C, the cell showed stable operation up to 20 C. The dopamine-heparin electrode showed stable performance up to 5 C, and showed a reasonably good cell performance of about 200 mAh g^−1^ even at 20 C. We attributed the enhanced rate properties of the heparin and dopamine-heparin electrodes to the improved physical properties of these binders, which helped to prevent the conducting agents and active materials from detaching from the electrodes even at high C rates. In addition, the functional groups in the heparin structure were thought to further provide a lithium transfer pathway capable of conducting Li^+^ ions^28,37–41^.

**Figure 5:**
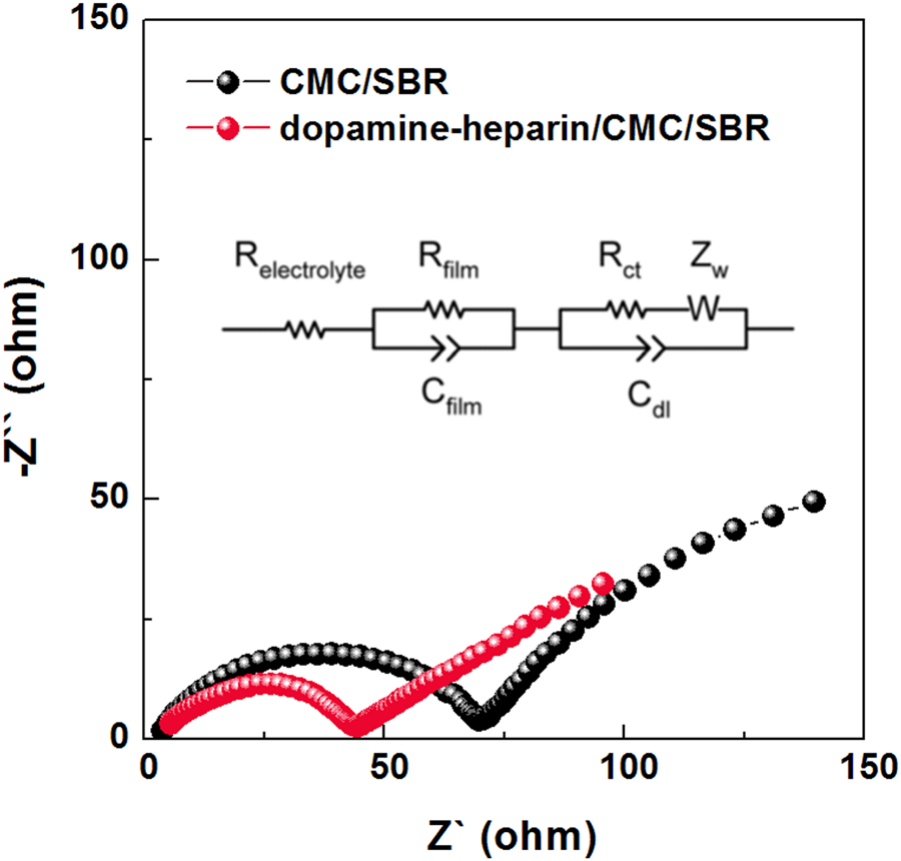
The EIS spectra for SiO_x_/graphite composite-based cells (black: CMC/SBR, red: dopamine-heparin/CMC/SBR).

Electrochemical impedance spectra (EIS) were also acquired for the electrodes with and without the dopamine-heparin, and the overall resistance on the electrode surface was found to be smaller when the dopamine-heparin was included (Fig. 5). Since there were no other factors besides the addition of dopamine-heparin, this result was thought to be due to the formation of a solid electrolyte interphase (SEI) layer. Surface analyses using SEM and XPS further confirmed these results (see below).

**Figure 6:**
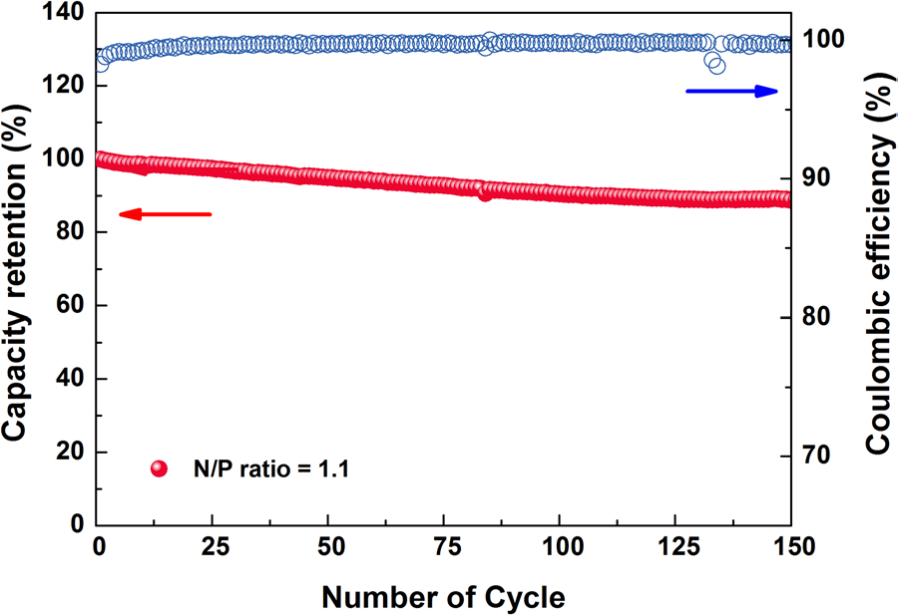
Cyclability results and Coulombic efficiencies of a full cell having the LiNi_0.6_Co_0.2_Mn_0.2_O_2_ cathode and SiO_x_/graphite composite anode including the dopamine-heparin/CMC/SBR binder.

Finally, a full cell, having a LiNi_0.6_Co_0.2_Mn_0.2_O_2_ positive electrode and the composite negative electrode containing the dopamine-heparin/CMC/SBR binder, was tested to evaluate the binder in a practical battery application (Fig. 6). To ensure reliable performance, one formation cycle was performed after the fabrication of the cell, followed by charging and discharging at 0.5 C between 3.0 and 4.1 V. Even though the initial high specific charging capacity of the full cell (Fig. S2), the obtained capacities at subsequent cycles suddenly fall because of the initial irreversible reaction of the negative electrode limiting the number of reversible lithium ions and the high kinetic resistance from the high amount of the active material causing high polarization. On the other side, an excellent cell performance, with retention of 92% of the initial performance at the 100^th^ cycle and of 89% of the initial performance at the 150^th^ cycle, was obtained, strongly suggesting that the proposed binder system has potential for use in practical Si-based lithium-ion batteries. In addition, the voltage profiles of the full cell (Fig. S2) showed 66.5% Coulombic efficiency at the pre-cycling step, which can be attributed to irreversible SiO_x_ conversion and SEI formation during pre-cycling. After the pre-cycling, however, the system required only two cycles to show >99% Coulombic efficiency, which was maintained for more than 100 cycles (Fig. 6).

### Morphological analysis

**Figure 7:**
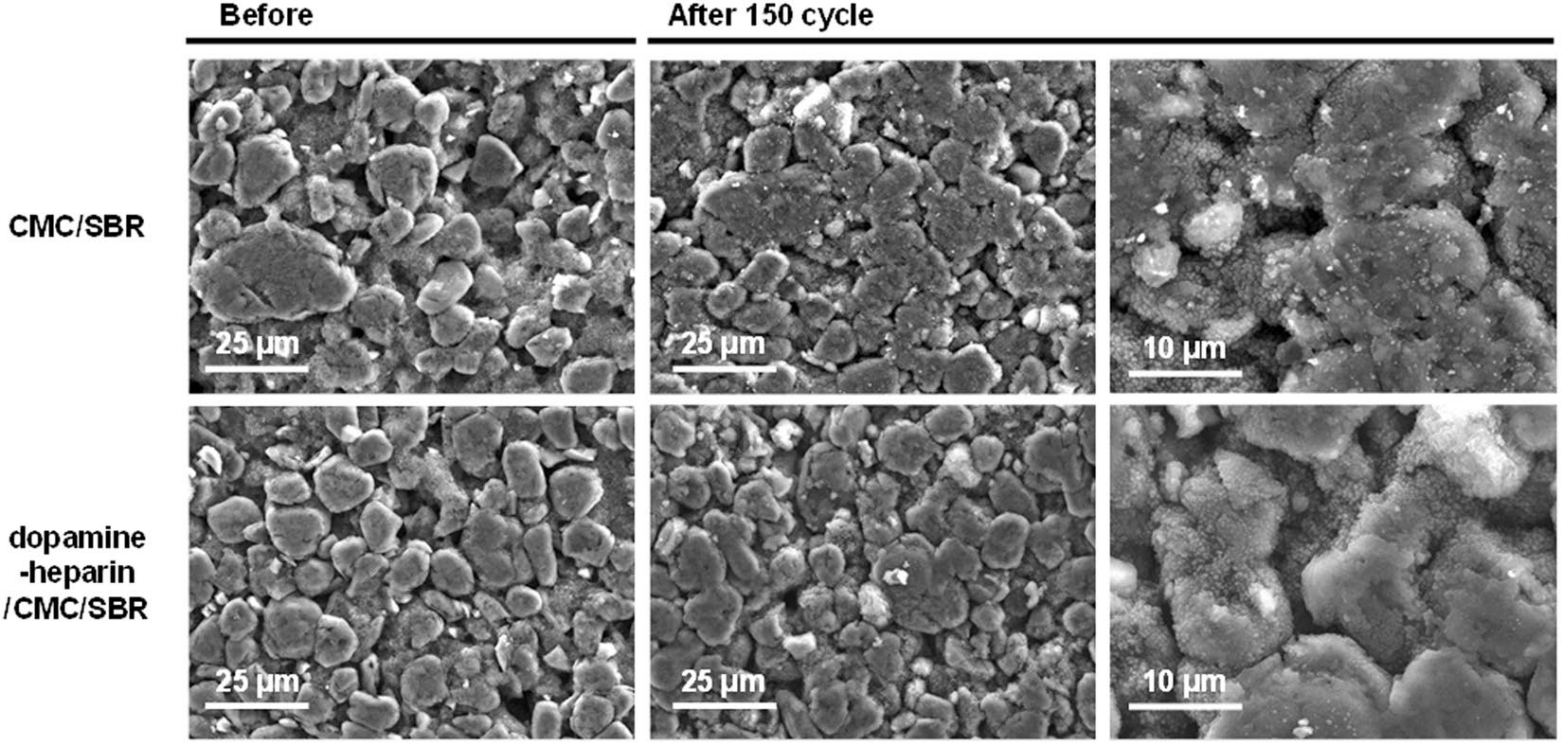
SEM images of the surface of the CMC/SBR-based and dopamine-heparin/CMC/SBR-based electrodes.

The CMC/SBR-based and dopamine-heparin/CMC/SBR-based electrodes were visualized before and after cycling using scanning electron microscopy (SEM), as shown in Fig. 7, in order to determine the effects of the binder material on the morphology of the electrode surface. The two electrodes showed similar surface morphologies immediately after fabrication, but quite different morphologies after 150 cycles, at which point the surface of the pristine CMC/SBR electrode was relatively thickly and unevenly covered by an SEI layer, while the surface of the dopamine-heparin/CMC/SBR-based electrode maintained a relatively clean, uniform and porous appearance.

Energy dispersive spectrometry (EDS) analyses of the prepared electrodes (based on analyses of mapped images of oxygen atoms, which originated from SiO_x_ and the polymer binder, and of silicon atoms, which originated from SiO_x_) showed that the materials comprising the electrodes were well dispersed for both electrodes irrespective of the addition of the dopamine-heparin to the CMC/SBR (Fig. S3). However, the EDS results showed that the portion of the weight of the CMC/SBR electrode due to oxygen was about 22% greater after cycling than before cycling, whereas the portion of the weight of the dopamine-heparin-based electrode due to oxygen was only about 20% greater after cycling (Fig. S4). Since changes in oxygen content as a result of cycling have been suggested to originate from the formation of the SEI layer and/or the decomposition of electrolytes, these results might indicate that less electrolyte decomposed when dopamine-heparin was present.

We also carried out XPS analyses of the surfaces of the CMC/SBR and dopamine-heparin/CMC/SBR electrodes. For the CMC/SBR electrode, the peak intensity corresponding to C_1s_ was much lower after 150 cycles than before cycling, whereas that for O_1s_ significantly increased, confirming the formation of a thick SEI layer caused by the side reactions of the carbonate-based electrolytes (Fig. S5). In contrast, in the case of dopamine-heparin/CMC/SBR electrode, the changes in the peak intensities of the C_1s_ and O_1s_ peaks were not large even after 150 cycles, suggesting that there was relatively little SEI formation^38,42^. The XPS results were found to be in good agreement with those of the SEM analyses.

## Discussion

We developed a new polymer binder system based on dopamine-grafted heparin (dopamine-heparin) as an additive to the CMC/SBR binder for SiO_x_/graphite composite electrodes in order to (1) produce physical crosslinking using acid-base interactions between heparin and CMC, (2) increase the ion conductivity using the sulfonate groups of heparin, and (3) strengthen the adhesion of the binder to the electrode due to the inclusion of dopamine. While the addition of heparin to the CMC/SBR binder (to convert commercialized CMC/SBR-based electrodes to the heparin/CMC/SBR-based electrodes) improved the physical properties of the electrodes and hence the overall performance of the corresponding cells, even better physical properties were achieved when dopamine-heparin was added (to produce dopamine-heparin/CMC/SBR electrodes). Also, while previously described electrodes could be made with only 3–5 wt.% SiO_x_ because of the increased failure of these electrodes with increasing SiO_x_ content due to side reactions of Si, we were able to achieve excellent cell performance for the dopamine-heparin/CMC/SBR-based electrode with SiO_x_/graphite composite active material containing up to 7 wt.% SiO_x_. Specifically, the resulting dopamine-heparin/CMC/SBR-based electrode displayed a specific capacity of 343 mAh g^−1^ and retention of 73.5% of the original capacity, and Coulombic efficiency of 99.7% after 150 cycles, together with a high capacity of 200 mAh g^−1^ even at 20 C. Furthermore, a stable cell performance with 89% retention at the 150^th^ cycle was achieved in a full cell test using this electrode. Overall, the inclusion of dopamine-grafted heparin not only enhanced the physical properties of the corresponding dopamine-heparin/CMC/SBR-based electrode but also its ionic conductivity, and hence demonstrated its potential for use in practical Si-based lithium-ion batteries.

## Methods

### Materials

Heparin sodium salt, N-hydroxysuccinimide (NHS), and dopamine hydrochloride were purchased from Sigma-Aldrich. N-(3-Dimethylaminopropyl)-N’-ethylcarbodiimide hydrochloride (EDC), sodium dihydrogen phosphate and sodium hydrogen phosphate were purchased from Alfa-Aesar. Dimethyl carbonate and electrolyte (1 M LiPF_6_ in ethylene carbonate/ethylmethyl carbonate (EC:EMC = 3:7 v/v) with 10 wt.% fluoroethylene carbonate (FEC)) were purchased from PanaxEtec. Silicon monoxide (SiO_x_) was obtained from Osaka Titanium Technologies. Carboxymethyl cellulose (CMC) was purchased from Nippon Paper Chemicals. Styrene-butadiene rubber (SBR) was purchased from ZEON.

### Synthesis of dopamine-grafted heparin 1

Heparin (1.0 g) was added to an aqueous phosphate buffer solution (PBS, 100 mL, pH = 6.0) in a round-bottom flask, and this mixture was stirred until a homogeneous solution was obtained. Thereafter, EDC (1.63 g, 8.5 mmol) and NHS (0.98 g, 8.5 mmol), dissolved in a PBS solution (pH = 6.0), were subsequently added to the heparin solution. Finally, to this mixture was added an aqueous solution of dopamine (0.5 g, 2.6 mmol) in PBS (pH = 6.0). The resulting mixture was stirred for 9 h at r.t. After completion of the reaction, the dopamine-grafted heparin was purified from the reaction mixture by subjecting this mixture to dialysis twice using a cellulose membrane (MWCO = 12 kDa, Sigma-Aldrich) in distilled water for 6 h, and the product was collected by freeze-drying.

The relative amount of dopamine in the dopamine-heparin **1** was calculated by performing quantitative analysis using UV-Vis spectroscopy (Fig. S1)^32^. Standard dopamine solutions were prepared for a calibration curve, and then the molar absorptivity was obtained from this calibration curve slope. Finally, the absorbance of the dopamine-heparin solution was measured, and the dopamine content was determined using the following Equation (1):

1 $${\rm{A}}={\rm{\varepsilon }}\mathrm{bc}$$

where A is the absorbance, Ɛ is the molar absorptivity, b is the path length of the sample, and c is the concentration of the sample.

### Fabrication of the SiO_x_/Graphite composite electrode

The composite electrode composition was 80:10:10 wt.% of active materials: conducting agent:polymer binder, with 7 wt.% of the active materials being SiOx and the remainder being graphite. The conducting agent is Super-P. The binder compositions tested were a 1:2 weight ratio of CMC:SBR and a 0.1:0.9:2 weight ratio of heparin(-dopamine):CMC:SBR. To make a SiO_x_/graphite composite electrode, we first mixed all of the solid materials, i.e., the graphite and SiO_x_ active materials, the conducting agent and CMC binder powder in a mortar based on the designed composition. This mixture of solids was combined with enough distilled water and a solution of SBR to make a slurry with the desired viscosity, which was coated onto a Cu foil. The as-formed electrode produced in this manner was dried in a convection oven at 80 °C for 30 minutes. The prepared electrode was made to have a mass loading of 5 mg cm^−2^. The prepared electrodes were dried under vacuum at 120 °C for 6 h to eliminate residual water. The theoretical capacity of the SiO_x_/graphite composite electrode was determined to be 450 mAh g^−1^ by weight ratio of active materials, whereas the capacities of graphite and SiO_x_ have been determined to be 372 mAh g^−1^ and 1500 mAh g^−1^, respectively.

### Characterization and Measurements

FT-IR spectra were recorded on a PerkinElmer Spectrum Two ATR spectrometer. UV-vis spectra were recorded on a PerkinElmer Lambda 365 spectrometer. Scanning Electron Microscopy (SEM) was performed using a JEOL JSM-7000F instrument. To see the surface morphology, the cycled Si electrodes were washed with dimethyl carbonate before being imaged. For evaluating the mechanical properties of the electrodes with various polymer binders, the prepared electrodes were each cut into rectangular shapes (1.2 cm × 3.0 cm) and attached to 12-mm-wide 3 M tape. The peel strength of each tested electrode specimen was then recorded with a universal testing machine (UTM, Shimadzu EZ-L) by pulling the tape at a constant displacement rate of 30 mm min^−1^.

### Electrochemical performances and failure mode analyses

To evaluate the electrochemical performances of the prepared electrodes, 2032-type coin cells were assembled in an Ar-filled glove box, using multi-layered porous PE/PP/PE as the separator, a lithium disc as the counter electrode, and 1 M LiPF_6_ in ethylene carbonate/ethylmethyl carbonate (EC:EMC = 3:7 v/v) with 10 wt.% fluoroethylene carbonate (FEC) (PanaxEtec) as an electrolyte. Galvanostatic discharge−charge cycling was then performed using a CPS-Lab battery cycler (Basytec) at 25 °C in a temperature-controlled chamber. All of the cells were repeatedly discharged to 0.005 V *vs* Li/Li^+^ and charged to 2.0 V *vs* Li/Li^+^ at a constant C-rate of C/20 lithiation (discharge)-C/10 delithiation (charge) in the first cycle and C/10 lithiation-C/10 delithiation in the next two cycles for pre-cycling, and then at C/5 lithiation- C/2 in the following cycles. Rate capability tests were carried out by repeating discharging to 0.005 V vs Li/Li^+^ and charging to 2.0 V vs Li/Li^+^. The pre-cycling was performed before the cyclability test, and the current for lithiation was fixed at C/20, and the currents for delithiation were varied from 1/2 to 20 C (C/2, 1 C, 2 C, 3 C, 5 C, 10 C, and 20 C). For postmortem analyses, the cycled cells were disassembled in an Ar-filled glove box and recovered electrodes were washed with dimethyl carbonate to eliminate residual electrolyte. Then, the surface morphologies of cycled electrodes were investigated by performing scanning electron microscopy (SEM, JEOL JSM-7800F). The EIS was measured at a frequency range of 10 mHz–100 kHz with an AC amplitude of 10 mV at 0.01 V.

### Full cell measurement

The full-cell was assembled using an N/P ratio of 1.1. The same SiO_x_/Graphite composite electrode was used for the negative electrode and fabricated 94:3:3 ratio of active materials:super-P:binder. The composite cathode electrode being fabricated with a 90:5:5 ratio of LiNi_0.6_Co_0.2_Mn_0.2_O_2_:super-P:PVdF were used. The full cells were repeatedly charged to 4.1 V and discharged to 3.0 V at a constant C-rate of C/10 for both the 1^st^ charge and discharge as a pre-cycling step and at C/5 of lithiation and C/2 of delithiation in the subsequent cycles. And 1 C was determined to be 170 mA per gram of the positive electrode material.

## Electronic supplementary material

Supplementary Information
