# Supplementary material for: Dopamine-grafted heparin as an additive to the commercialized carboxymethyl cellulose/styrene-butadiene rubber binder for practical use of SiOx/graphite composite anode
Source: Sci Rep. 2018 Jul 27;8:11322. doi: 10.1038/s41598-018-29705-y (PMC6063964; doi:10.1038/s41598-018-29705-y)
Supplement: Supplementary file 1 — Supplementary Information [file 41598_2018_29705_MOESM1_ESM.docx]

**Dopamine-grafted heparin as an additive to the commercialized carboxymethyl cellulose/styrene-butadiene rubber binder for practical use of SiO_x_/graphite composite anode**

Kukjoo Lee, ^1,2^ Sanghyun Lim, ^1,2^ Nakgyu Go,^3^ Jaemin Kim,^3^ Junyoung Mun, ^3^ and Tae-Hyun Kim,^1,2,^*

^1^Organic Material Synthesis Lab. Department of Chemistry

^2^Research Institute of Basic Sciences, Incheon National University, 119 Academy-ro, Songdo-dong, Yeonsu-gu, Incheon 406-772, Korea

^3^Department of Energy and Chemical Engineering, Incheon National University, 119 Academy-ro, Songdo-dong, Yeonsu-gu, Incheon 406-772, Korea

^*^Corresponding Author, [tkim@inu.ac.kr](mailto:tkim@inu.ac.kr) (T.-H. Kim)

Figure S1. UV-Vis spectra the dopamine solution under various concentration (a), calibration curve between molarity and absorbance (b), heparin and dopamine-heparin spectra and absorbance value (c).

Figure S2. The charge-discharge galvanostatic curves of the LiNi_0.6_Co_0.2_Mn_0.2_O_2_-SiO_x_/graphite full cell with pre-cycling step.


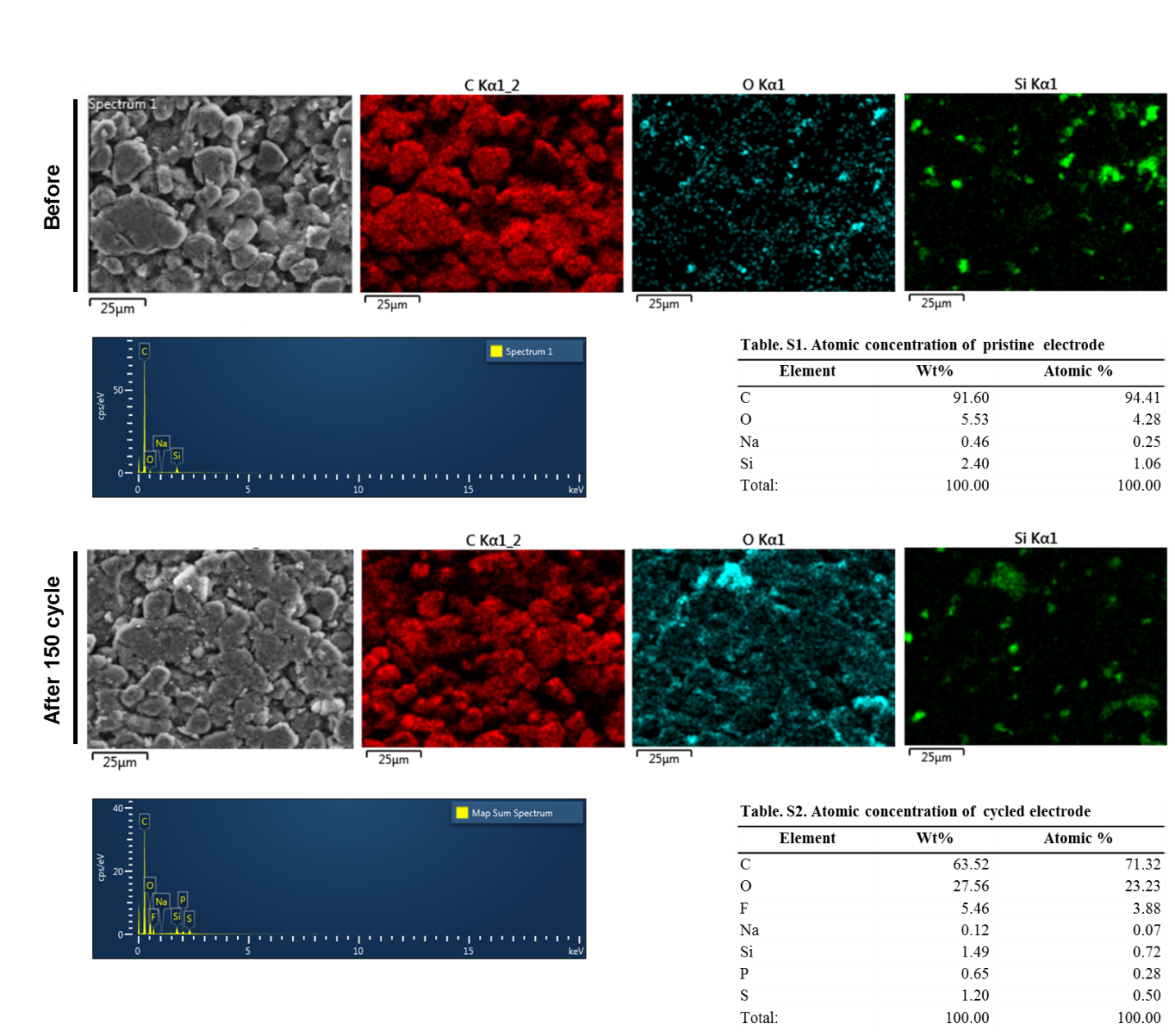


Figure S3. EDS analysis result and mapping image of pristine and cycled CMC/SBR electrode.


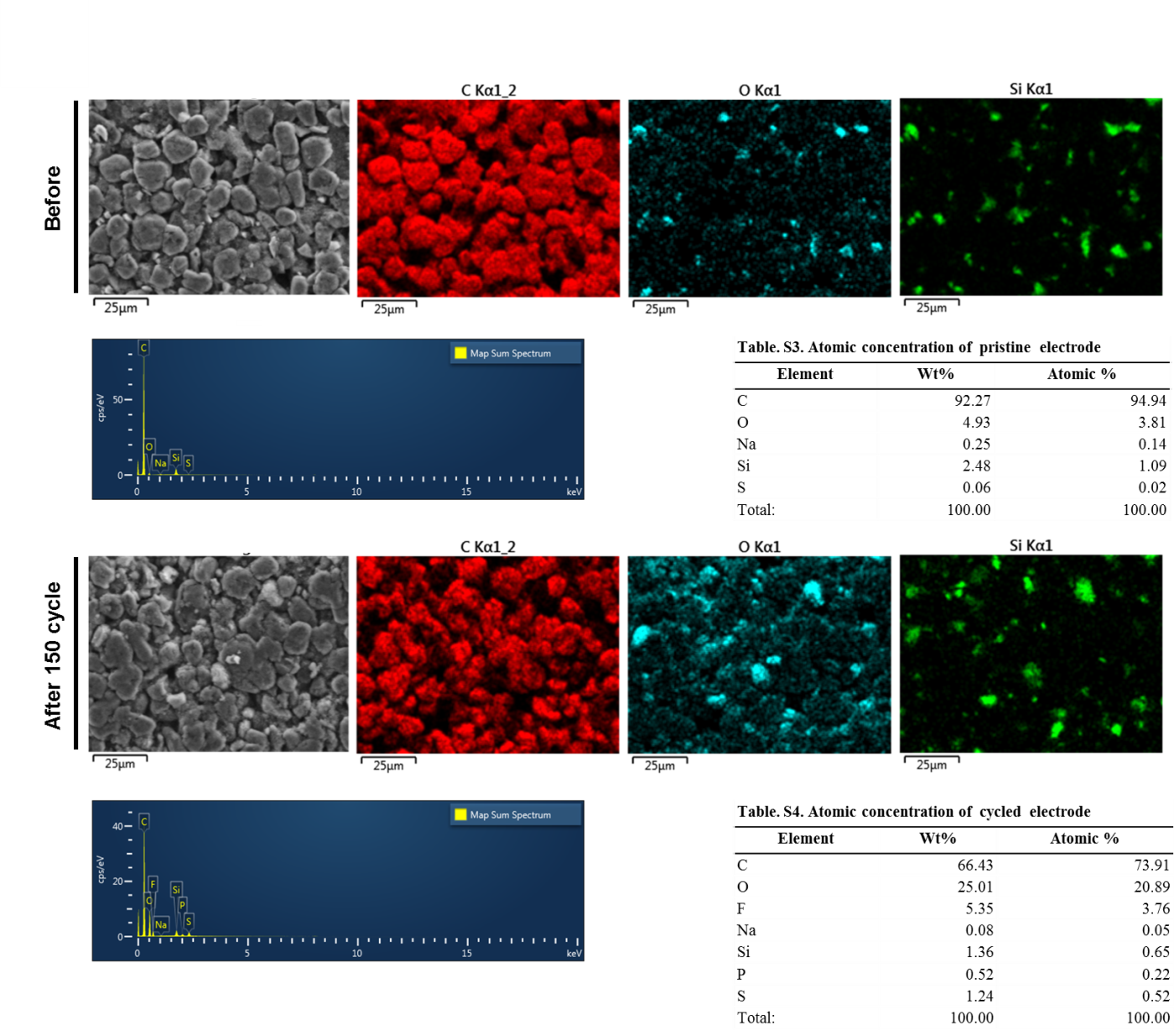


Figure S4. EDS analysis result and mapping image of pristine and cycled dopamine-heparin/CMC/SBR electrode.

Figure S5. XPS spectra of electrodes made of CMC/SBR binder (a and b) and dopamine-heparin/CMC/SBR binder (c and d): C_1s_ (a and c) and O_1s_ (b and d).
